# Implementing climate menu labels in university settings: a narrative review

**DOI:** 10.3389/fnut.2025.1619842

**Published:** 2025-11-21

**Authors:** Mei-Li Hey, Elizabeth Crespi, Ariana Yett, Daphene Altema-Johnson, Julia Wolfson, Rebecca Ramsing

**Affiliations:** 1 Center for a Livable Future, Department of Environmental Health and Engineering, Johns Hopkins Bloomberg School of Public Health, Baltimore, MD, United States; 2 Department of International Health, Johns Hopkins Bloomberg School of Public Health, Baltimore, MD, United States; 3 Department of Health Policy and Management, Johns Hopkins Bloomberg School of Public Health, Baltimore, MD, United States

**Keywords:** climate, carbon emissions, label, university dining, greenhouse gases

## Abstract

Global food systems are a major contributor to climate change, accounting for more than 30% of greenhouse gas emissions (GHGEs). This review synthesizes current evidence on the potential of climate labels (which we define as labels attached to menus or food items with climate impact information) in university dining settings to encourage climate-friendly food decisions. In April 2024, we searched Scopus, ProQuest, Web of Science, and PubMed using developed terms related to climate labels and universities. We identified 280 articles and narrowed down the review to 14 articles based on study setting, language, and scope. Of the 14 studies, 86% (12 articles) observed a decrease in the consumption of foods researchers classified as high emissions, 60% (8 articles) observed a decrease in the consumption of foods classified as medium-emission foods, and 63% (9 articles) observed an increase in the consumption of foods classified as low-emission food. Effects varied by gender and age, with women appearing to experience a greater response to the climate labels, but no observable differences were evident by ethnicity or socio-economic class. Studies that supplemented climate labeling initiatives with sales promotions or resources encouraging consumers to conduct their own emission research also saw favorable results. The studies suggest a small yet detectable shift in consumer behavior in response to climate labels in university dining settings; however, further research is needed on: (1) improving climate label effectiveness, (2) the effect of climate labels among different demographic attributes (e.g., income, ethnicity), and (3) the long-term and spillover effects of the labels on the healthiness of consumer diets, and institutional sourcing practices.

## 1 Introduction

Climate change is increasingly recognized as one of the most pressing issues of our time, with severe implications for the environment and health. Food systems are both impacted by and contribute to climate change. An estimated 34% (18 GT) of total annual global greenhouse gas emissions (GHGEs) are attributed to food supply chains (1).^1^ Substantial reductions in food system GHGEs are needed to limit global average temperature increases to 1.5–2 °C (3) as outlined in the Paris Agreement, and to remain within food system planetary boundaries (4, 5).

The amounts of GHGEs generated from food supply chains vary by several orders of magnitude across food groups, for example, a mean of 1.0 kg CO_2_e^2^ per 100 g peas compared to 99.5 kg CO_2_e per 100 g beef (6). Thus, diets can produce widely different amounts of GHGEs depending, in large part, on the amounts and composition of animal source foods included (7). Compared to the status quo, a shift toward diets with lower amounts of GHGE-intensive foods, such as red meat, has the potential to reduce food system GHGEs by 29–56% by 2050 (5).

Over the last few decades, labels highlighting the climate impacts of products (usually the item's GHGEs or other climate indicators) emerged as a key strategy to shift consumers toward more sustainable behaviors and help meet climate mitigation goals (8–10). Prior evidence supports the effectiveness of labels in shifting consumer behavior (11). For example, a 2018 meta-analysis found that nutrition labeling reduced consumer energy (kilocalorie) and fat consumption and increased vegetable consumption (12). The U.S. Energy Star label on appliances reduced CO_2_e emissions associated with household energy use in 2020 by an estimated 400 metric tons (5% of US total GHGEs) (13).^3^ Even in cases when consumers have little to gain personally from a more environmentally friendly choice, as in the example of dolphin-safe labeling on tuna, labeling can shift consumer behaviors (14). Some consumers are also willing to pay more for environmentally labeled food products (15). A 2024 review found that environmental labels on food are particularly effective for individuals who clearly understand the labels or are already concerned about the environmental impact of their food (16). A 2022 Intergovernmental Panel on Climate Change report similarly concluded that food labeling can be effective in shifting behaviors that lead to positive environmental impacts (17).

Previous studies have concluded that not all labels are equally effective in changing consumer behavior, and certain design characteristics work better than others for promoting climate friendly food options (18, 19). These studies vary in setting, label design, classification of GHGE intensity, and messaging about the label and its purpose (20–22). One popular design for climate labels, for example, is a traffic light label (TLL), a type of ordinal rating label which uses red, yellow, and green labels corresponding to high, medium, and low GHGEs, respectively (20–22). Labels based on traffic light colors that are simple and easy to read have been shown to be particularly effective at changing consumer behavior (19).

The potential effectiveness of labeling interventions in influencing consumer behavior can be attributed to two different known mechanisms. First, consumers are often unaware of the environmental costs of food production (10, 20). Providing clear information about the climate footprint of food items enhances consumer awareness and understanding of the environmental impact associated with their choices (23, 24), thus allowing consumers to compare products and make more informed decisions aligned with their environmental values (9, 23). Second, labels can serve as timely reminders that prompt consumers to consider sustainability factors in their decision-making process, without restricting their freedom of choice (25). Together, these mechanisms underscore the potential of labeling interventions to foster significant shifts toward sustainable consumption patterns that contribute to broader environmental goals.

While climate labels are used in a range of contexts, for this narrative review, we focus on studies on the use of climate labels in university dining settings, as they offer a unique opportunity to explore behavioral and social dynamics in a controlled yet real-world environment. Universities can operate as living-laboratories that test strategies and facilitate ongoing implementation studies. Universities also serve as environments in which students can influence each other thorough social interactions and group dynamics, which allows researchers to study how climate labeling affects both individual and group behaviors and norms within a community. Finally, behaviors formed during college can persist later in life (26, 27), presenting an opportunity to instill long-term habits in diners related to sustainable eating.

To compare studies on the design, implementation, and effect of climate labels in university settings, we conducted a narrative review of the existing literature. There is a range of existing research on climate labels in university settings which, to the best of our knowledge, no other literature review (systematic or otherwise) has summarized to date. Thus, the aim of this study is to address this gap in the current literature, and to contribute to greater understanding of the potential effects of climate labels, best practices in their implementation, and potential areas for future research on climate labels in university settings.

## 2 Methods

We searched Scopus, ProQuest, Web of Science, and PubMed databases in April 2024. We used the following search string, developed in consultation with a university librarian: [(“climate^*^” OR “carbon^*^”) AND (“food^*^” OR “dine” OR “dining” OR “dined” OR “meal^*^”) AND (“college^*^” OR “universit^*^” OR “graduate student^*^” OR “undergraduate student^*^”) AND (“label^*^” OR “nudg^*^” OR “information disclosure^*^” OR “intervention^*^”)]. The search methodology resulted in 451 articles. After removing duplicates identified by Covidence, two researchers reviewed the remaining 280 papers to determine eligibility based on the following inclusion criteria:

1. Study is available in English;
2. Study includes primarily university students in the sample and/or takes place in a university setting (e.g., dining hall, cafeteria, or a campus grocery store);
3. Study tests perceptions, effectiveness, or implementation of a menu and/or food item label representing metrics specific to climate change (e.g., carbon or GHG footprint);
4. Study presents new data (e.g., not commentaries).

**Figure 1:**
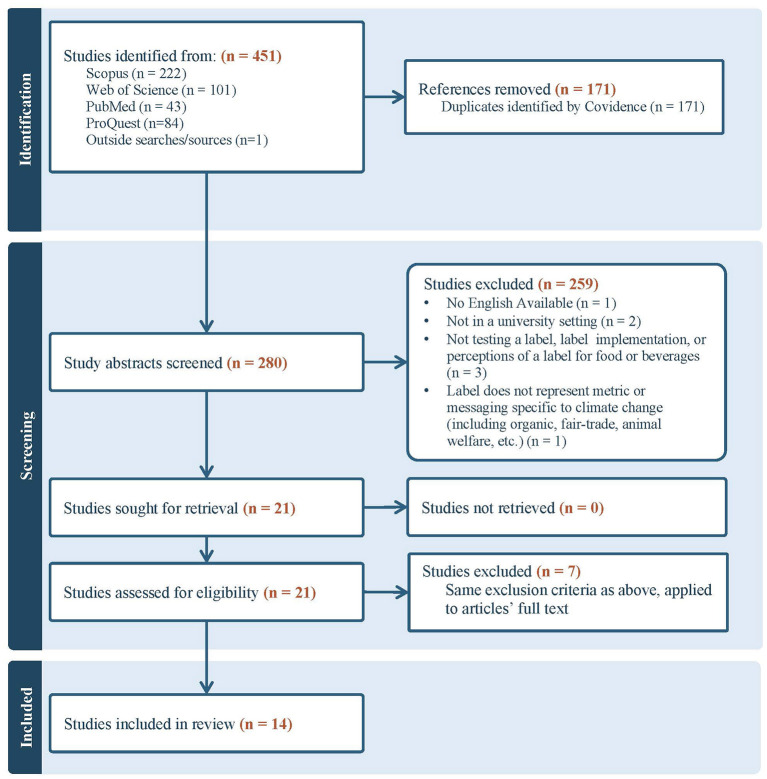
PRISMA diagram.

Twenty-one full-text articles were assessed for eligibility, with seven subsequently excluded. Figure 1 depicts a PRISMA diagram that outlines this study selection process. This process yielded a final 14 studies from which relevant content (Table 1) was then extracted.

**Table 1:** Summary of studies.

**Author**	**Year**	**Country**	**Study time frame**	**Study type**	**Type of data collection**	**University setting**	**Sample size**
Kimura et al. (39)	2010	Japan	2009	Cross-sectional	Survey based on a hypothetical scenario	Simulation designed to replicate food purchases	Survey: *n* = 151
Cholette et al. (40)	2013	United States	April 2010	Cross-sectional	Survey based on a hypothetical scenario	Simulation designed to replicate food purchases	Survey: *n* = 428
Brunner et al. (30)	2018	Sweden	Two phases from February 1st to March 11th (control) and April 11th to May 27th of 2016 (exposed)	Quantitative pre-/post- intervention studies supplemented by an education and outreach campaign with poster and flyers	Sales data on number/type of dishes sold	Cafeteria with pre-set meal options	Control & Treatment: 300–600 servings
Slapø and Karevold (32)	2019	Norway	Not reported	Quantitative pre-/post- intervention studies with no control supplemented with posters placed in public locations.	Sales data on the number/type of dishes sold	Cafeteria with pre-set meal options	177
Zhao et al. (37)	2020	China	2015–2016	Quantitative intervention study; auction experiment; interviews	Sales volume; price assigned during bidding; qualitative data	Campus grocery store; simulated auction	Interviews: *n* = 24; Auction Experiment: *n* = 50; Sales experiment: *n* = 192.
Piester et al. (35)	2020	United States	Not reported	Single-timepoint randomized control trial	Survey data, sales data on the number/type of dishes sold	Campus cafeteria	Study 1: 176; Study 2: 228
Larner et al. (31)	2021	England	Label intervention: May–June 2019.	Quantitative pre-/post- intervention studies with no control supplemented with explanatory posters	Sales and purchasing data on the number/type of dishes sold	Four different campus dining spots: salad bar, coffee shop, café, and restaurant	Survey: *n* = 643; Interviews: *n* = 66
Malan et al. (28)	2022	United States	Fall 2019	Quantitative pre/post intervention study with non-randomized controls supplemented by posters, table tents and physical signs explaining the study	Sales data on the number/type of dishes sold	Campus quick service restaurants	Intervention = 325,250 entrees; Control = 320,572 entrees
Castellanos et al. (36)	2022	United States	Study does not mention specific dates, only indicates that CO_2_ emissions data was calculated from 6 months of sales data.	Qualitative post-intervention interviews supplemented by an education campaign on sustainability.	Sales data on dining halls; descriptive qualitative data	Campus dining hall	Surveys: *n* = 41; Interviews: *n* = 8
Isham et al. (38)	2022	England	Not reported	Cross-sectional online survey	Survey based on a hypothetical scenario	Simulation designed to replicate liking/willingness to pay	Survey: *n* = 100
Lohmann et al. (29)	2022	England	October 7-December 8, 2019 and Jan 13-March 15 2020	Quantitative pre/post intervention study with non-randomized controls	Individual-level, meals sales data	Campus cafeteria	Treatment: 39,053 purchases; Control: 42,348 purchases
Suchier et al. (41)	2023	France	Not reported	Single-timepoint randomized control trial	Sales data per “shopping basket”	Simulated campus grocery store	Survey: *n* = 288
Sun et al. (33)	2024	China	Control: 12/9–12/31/2020. Treatment: 3/8–3/31/2021	Quantitative pre-/post- intervention studies	Sales data on number/type of dish sold	Campus canteens	Control = 8,755; Treatment = 9,492 participants
Thamer et al. (34)	2024	Germany	Not reported	Quantitative pre-/post- intervention studies with no control	Reported number/type of dishes sold	Campus cafeteria	Participants = 129; Meals = 645

## 3 Results

### 3.1 Overview of included studies

Among the studies reviewed, seven were conducted in Europe, four in the United States, and three in Asia (Table 1). All studies were published between 2010 and 2024. Across all studies, the primary objective of labeling was to communicate climate-related information to consumers. Of the 14 studies, 10 implemented actual climate label interventions in campus dining facilities. These 10 studies were conducted in a number of different ways: seven studies compared pre-/post-intervention data with (*n* = 2) (28, 29) or without (*n* = 5) (30–34) control groups; one study conducted a single-timepoint randomized controlled trial comparing treatment and control groups (*n* = 1) (35), another exclusively focused on collecting qualitative data post-intervention (*n* = 1) (36), and the last real-world study collected quantitative data pre-intervention, and supplemented this with a live auction experiment and qualitative interviews (*n* = 1) (37). The remaining four studies included this review utilized online survey experiments, focused on consumer willingness to pay (38, 39) or hypothetical purchase behaviors (Table 1) (40, 41).

The 10 intervention studies also had varying study lengths and follow-up periods with consumers after labeling. Some studies were sustained for a few days (31), while others several months (29). In addition, some of the studies had a follow-up period several months after the intervention (Table 1) (37).

### 3.2 Climate label naming, design, placement, and implementation

Across the 14 studies, the climate labels were used to convey information about the GHGE intensity of the menu items. However, the label, design, placement, and implementation all differed. In addition to referring to the labels as “climate labels,” the authors also called them carbon, environmental, or eco labels. Beyond just naming, the specific metrics, calculation methods, groupings, and label designs also varied across studies (Table 2).

**Table 2:** Label definition, design, and placement in included studies.

**Author and publication year**	**Label metric**	**Label metric calculation**	**Label categories**	**Label design (from study)**	**Label placement**
Kimura et al. (39)	CO_2_ emissions per product in grams	Fictional CO_2_ emission scores estimating industry average for each food product category	Low: 10 g below average CO_2_ emissions for product category Medium: average CO_2_ emissions High: 10 g above average CO_2_ emissions	The black and white image depicts the CO_2_e of a product in a circle (e.g. “60g” for the low CO_2_e condition), with option to click view a breakdown of the carbon emissions. After a user clicks, the product's carbon emissions (by %) is shown, including growth of ingredients, production, packing, transportation, and waste.	Underneath a picture of the food item on the online survey
Cholette et al. (40)	CO_2_e emissions of apple	No formal calculations	Low CO_2_e footprint	No design created	Hypothetically on the apple
Brunner et al. (30)	kgCO_2_e emissions of entire meal	Emissions data from Bryngelsson 2016 (51), Winther 2009 (52), Head 2011 (53)	Green: ≤ 0.9 kg CO_2_e Yellow-orange: 0.9–2 kgCO_2_e Red: >2 kgCO_2_e	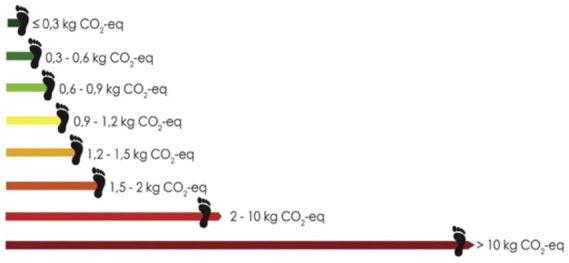	Underneath each dish on online and printed menus
				[A breakout of the different emissions ranges associated with specific dishes]. Reproduced from (30) with permission from Elsevier.	
Slapø and Karevold (32)	CO_2_ footprint of meal	No formal calculations	Low: vegetarian Medium: fish High: meat other than fish	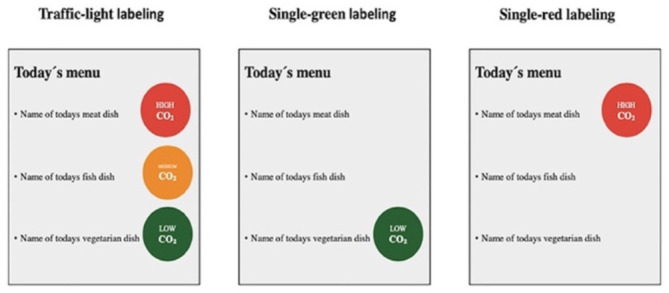	To the left of the meal on the menu board
				[A snapshot of the different ways climate labels was used in the study: TLL labeling, single-green labeling, and single red labeling]. Reproduced from (32), licensed under CC BY 4.0.	
Piester et al. (35)	Sustainability of entire menu item based on carbon, nitrogen, and water footprints	Methods from Leach 2016 (54)	Five groups representing relative environmental impact of menu item	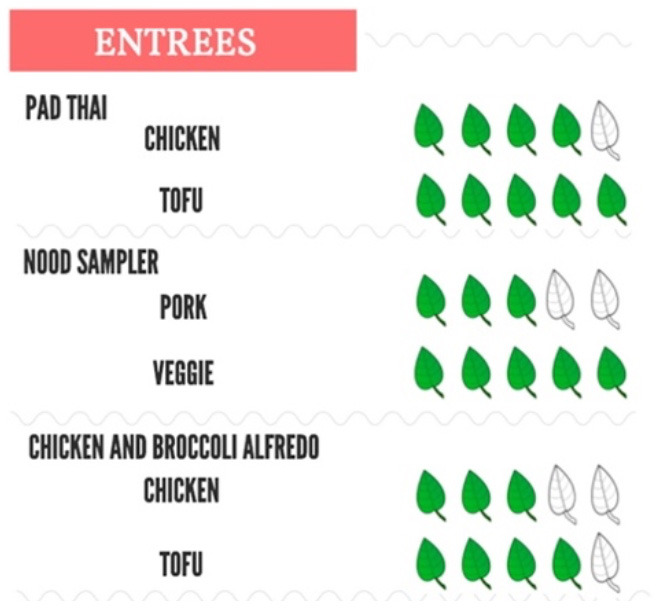	Study 1: To right of meal name on menu. Study 2: Had a separate screen with picture of veggie burger and label.
				[Example of a menu with different entrees containing different sustainability scores designated by the different numbers of colored leaves. The more leaves = better environmental impact]. Reproduced from (35) with permission from Elsevier.	
Zhao et al. (37)	CO_2_e emissions of milk	Emissions data from Zhao 2012 (55) and Zhao 2018/8/25	Low emissions: 200 g CO_2_e emissions	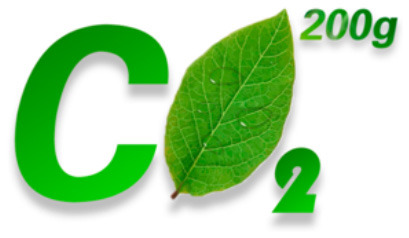	On the milk packaging
				[The letter C, next to a leaf with the number 200 g above it]. Reproduced from (37), licensed under CC BY 4.0.	
Larner et al. (31)	kg CO_2_e of prominent ingredients	Methods from Graham 2018 (56); Emissions data from Clune 2017 (57).	Low emissions: all ingredient kgCO_2_e emissions <4.44	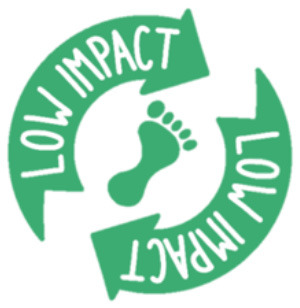	Varied based on venue (e.g., on ‘Low impact foods below' magnet in salad bar, on ‘Milk guide' in café and restaurant)
				[An example of a low impact label: a footprint inside a cycle diagram]. Reproduced from (31), licensed under CC BY 4.0.	
Castellanos et al. (36)	kgCO_2_e emissions per kg of meal	Emissions data from Food Carbon Emissions Calculator	Green: bottom 50% kgCO_2_e emissions Yellow: 50–75% Red: top 25%	Menu items or ingredients at the build-your-own meal stations highlighted in colors corresponding to kgCO_2_e	On the menu
Isham et al. (38)	Ecological or health impact	Research on existing products in the same category	Eight groups	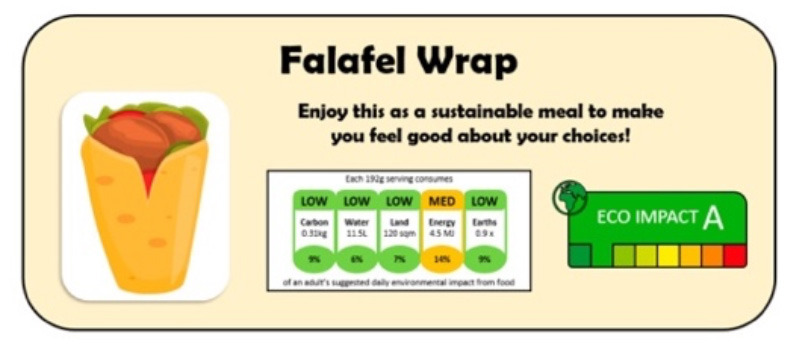	Next to a picture of the food item on the survey
				[A climate label for falafel detailing it's eco impact score, and the carbon, water, land, energy, and earth impact of production]. Reproduced from (38), licensed under CC BY 4.0.	
Lohmann et al. (29)	gCO_2_e per 100 g of meal	Emissions data from Clune 2017 (57), Poore 2018 (6), Hilborn 2018 (58), adapted to British food procurement system	Quintiles (per 100 g of meal, lowest 20% had gCO2e <150 and highest 20% had gCO2e >800)	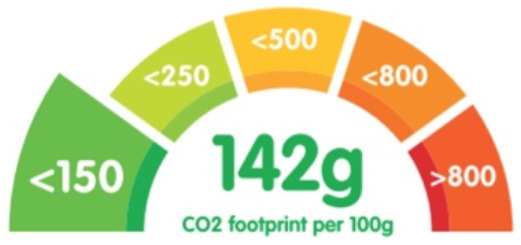	Directly above the meal where it is served
				[Emissions data for a specific meal, depicting the CO_2_e and the color coded quantile the emissions fall into]. Reproduced from (29), licensed under CC BY 4.0.	
Malan et al. (28)	Percent kgCO_2_e contribution of main ingredients to daily footprint of reference diet	Methods from Leach 2016 (54)	Low emissions: ≤ 25% contribution to CO_2_e of reference diet	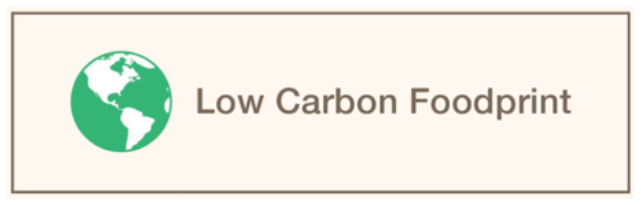	Next to menu items on online and in-person menus
				*[Depiction of the low carbon footprint label]*	
Suchier et al. (41)	gCO_2_e per 100 g serving	Emissions data from Tesco 2012 (41) and French Agency for Ecological Transition ADEME 2020	Inter-TLL: gCO_2_e of all products in the shop or in the same food category Intra-TLL: Low: bottom third Medium: middle third High: top third	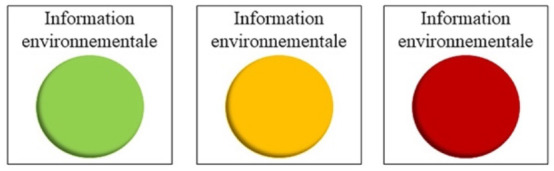	In the bottom left corner of the icon for each food item on the online store
				[Depiction of the TLL used for both inter and intra-TLL analysis]. Reproduced from (41), licensed under CC BY-NC 4.0.	
Sun et al. (33)	kgCO_2_e of dumplings (excluding salts, MSG, soy sauce, vinegar, spices, cooking wine)	Emissions data from Xu 2016 and Clune 2017 (57)	Low: bottom 25% kgCO_2_e Medium: 25-75% kgCO_2_e High: top 25% kgCO_2_e	The label depicts the carbon footprint of 5 different dishes arranged from lowest to highest carbon footprint per kcal. Each number next to the dish is color-coded green, yellow or red based on the carbon footprint. A horizontal scale at the bottom of the label with a color gradient from green to red is provided to show the magnitude of the difference in the carbon footprint across all 5 dishes.	On the menu, which was next to the point of sale
Thamer et al. (34)	Presence or absence of meat	No formal calculations	Single label for vegetarian items	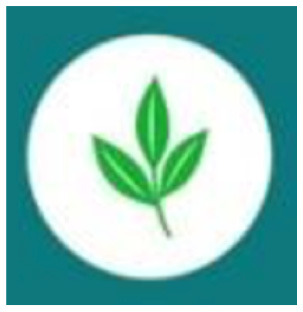	Directly above meal and salad bar and next to items on menu blackboard
				[Example of the vegetarian label applied to menu items]. Reproduced from (34), licensed under CC BY 4.0.	

Nine of the 14 studies calculated the GHGE intensity of each food item using pre-existing emissions intensity data and/or methodologies from academic papers (*n* = 7) (28–31, 33, 35, 37) or other online sources (*n* = 2); e.g., Food Carbon Emissions Calculator, French Agency for Ecological Transition (36, 41). For these nine studies, the label often depicted the g or kg CO_2_e associated with the prominent ingredients in a meal, the entire food item or meal, per 100 g of the meal, or as a percent contribution to the CO_2_e emissions of healthy reference diet (*n* = 8) (28–31, 36, 37, 41). One study also incorporated nitrogen and water footprints in the label metric (35). The five studies which did not calculate the GHGE intensity associated with food items, instead labeled dishes based on the general meal type (e.g., vegetarian/non-vegetarian or vegetarian/fish/meat) (32, 34), randomized use of the label on a uniform product (40), or used fictional CO_2_e emissions (39) and ecological impacts (38) based on data collected on other products in the same category (Table 2).

Across the board, the studies were heterogeneous in how the labels categorized and depicted GHGE intensity. Five studies only differentiated food items classified as having low carbon emissions, (31, 34, 37, 40) five studies created three categories (low, medium, high) (28, 32, 33, 36, 39, 41) and four studies depicted a scale (low-high) on their label to convey GHGE intensity (29, 30, 35, 38). Specific strategies for determining cutoffs for the groupings varied but were often based on GHGE intensity of the food/ingredient being labeled compared to all food items or meals in the study. Throughout this review, we use the authors' classifications of labels for our reporting.

Of the 14 studies, 12 employed a color scheme to visually communicate GHG emissions. For the five studies with a single low emissions label, green was generally used as the label color (31, 34, 37), though one involved no color scheme (40). For studies with three or more emissions groups (29, 30, 32, 33, 36, 38, 41), a scale of green for low emissions to red for high emissions items was generally used; however, two used just green (28, 35) and one (40) did not include a color scheme (Table 2).

For the 10 intervention studies (28–37), labels were displayed physically on menus (28, 30, 32, 33, 35, 36), in the immediate area near where the food was served (29, 34), on the food item packaging (37), or next to food items on a digital menu (35). For the hypothetical survey studies, labels appeared near a photo of the food item (38, 39, 41) or were described as placed on the food item itself (e.g., a produce sticker) (Table 2) (40).

Five of the 10 intervention studies incorporated additional interventions alongside label implementation for the purpose of enhancing label effectiveness (28, 30–32, 36). The supplemental interventions were often informational posters displayed at strategic locations within the dining hall to explain the labeling system and/or sustainability information (e.g., the importance of reducing GHGEs) (28, 30–32). Castellanos et al. introduced a secondary intervention involving an educational program aimed at enhancing general sustainability knowledge, though this was the only study of these five studies that found no behavioral effect from the labels (36). Notably, only the Castellanos et al. study explicitly discussed fidelity, which they use as an implementation outcome to measure whether the intervention was delivered as intended. Based on the results of our narrative review, there are research gaps in understanding the effectiveness of combining labels with other interventions in university dining halls.

### 3.3 Climate label impacts

Of the 14 included studies, most tested the effectiveness of climate labels in changing real-world consumer behavior (*n* = 10) or hypothetical behavior changes (*n* = 3). Four of the 13 studies also tested group-level differences in climate label effect. The remaining study tested group-level differences in consumers' hypothetical responses to climate labels (Table 1). Of the 13 included studies that assessed whether climate labels change consumer behavior, 11 found them to be effective in at least one circumstance; the remaining two, one post-intervention qualitative study of eight students (36) and one online hypothetical willingness-to-pay (WTP) study of 100 students (38), found no effect. The following sections describe the studies and the corresponding results in further detail (Table 3).

**Table 3:** Summary of results and label impact (*n* = 14).

**Author**	**Year of publication**	**Key results**	Label impact^*^	**Were effects heterogenous? If not—what group experienced a higher impact?**
Kimura et al. (39)	2010	Accessibility of information combined with carbon emissions can affect consumer attitudes. The climate label creates value for the product only when participants actively search for the information.	+	Not assessed
Cholette et al. (40)	2013	Female and older individuals were more likely to select climate-friendly food regardless of their financial situation. The probability of an individual choosing a climate-friendly option regardless of their financial situation was higher among white students than from other ethnic groups. As respondents grow older and gain wealth, they tend to choose climate friendly options more frequently.	N/A	Female, white, and older participants
Brunner et al. (30)	2018	Sales of green labeled (low emission) meat dishes experienced the only significant change, increasing by 11.5% compared to the control phase.	+	Not assessed
Slapø and Karevold (32)	2019	TLL label: Sales of red-labeled dishes fell 9%; green- and yellow- labeled dish sales did not change. Single green or red label: No change in sales for any dish.	+	Not assessed
Piester et al. (35)	2020	A higher percentage of women (females) in the intervention (vs. control) group purchased climate friendly meals (>4 leaves) (38% vs. 6%) and vegetarian items (26% vs. 7%); no difference observed for men.	+	Females
Zhao et al. (37)	2020	Depending on price, 56–79% of participants purchased carbon labeled over non-carbon labeled milk; the mean percentage of bidders was 16% higher for carbon-labeled vs. non-carbon labeled milk.	+	Not assessed
Larner et al. (31)	2021	Medium and low emissions ingredient use in the salad bar, dairy milk use in the cafe, and low impact sales values in the burger bar decreased. The use of oat milk in the cafe and medium and high impact sales value in the burger bar increased. With a label and sales promotion, beef burger sales fell 35.5%, chicken burger sales increased 20.3%, and meat-free burger sales increased 15.1%.	+	Not assessed
Lohmann et al. (29)	2022	The market share of high-impact meals fell 2.7%, medium-impact meals increased 2.7%, and low-impact meals did not change; the average meal footprint was reduced 27 g CO_2_e per 100 g serving.	+	Participants following high-carbon footprint diet
Malan et al. (28)	2022	The proportion of low-CO_2_e entree sales increased 54%, medium-CO_2_e increased 8%, and high-CO_2_e decreased 9%. The average entree footprint decreased 117 g CO_2_e	+	Not Assessed
Isham et al. (38)	2022	Environmental framing on food labels does not have a significant effect on consumer choices or willingness to pay, but higher levels of positive wellbeing is associated with a greater willingness to pay for climate friendly options.	0	Participants with higher levels of positive wellbeing
Castellanos et al. (36)	2022	Sustainable diet attitudes were negatively correlated with animal product consumption. Students felt the intervention increased awareness on sustainable eating but reported no change in their behavior.	0	Not assessed
Suchier et al. (41)	2023	With TLLs, purchasing of red-labeled products lower (Intra: −18%, Inter: −11%) and green-labeled products higher (Intra: +21%, Inter: +14%); no change for yellow-labeled products. The kgCO_2_ of baskets was lower for Intra- and Inter-TLL vs. the controls (Intra-TLL: 2.2, Inter-TLL: 2.2, Control 1: 3.1, Control 2: 2.8).	+	Not assessed
Sun et al. (33)	2024	Dumpling sales and average sales per transaction decreased and number of transactions increased for red- vs. yellow- labeled dumplings; no change for green-labeled dumplings.	+	Females
Thamer et al. (34)	2024	Meat demand was reduced (5–7%) only when the labeling was paired with an email encouraging reflection on the label; the effect waned when intervention ended.	+	Not assessed

^*^Note: “+” indicates a positive behavior change in the desired direction was observed for any subsection of the study: a decrease in the consumption of high emission foods OR increase in the consumption of low emission foods is reported (11 papers); “–” indicates a negative behavior change: an increase in the consumption of high emission foods OR a decrease in the consumption of low emission foods is reported (0 papers); “N/A” indicates that labeling effects were not assessed (0 applicable papers), and “0” indicates no effect on consumer behavior detected (2 applicable papers).

#### 3.3.1 Intervention studies examining the effect of climate labels on real world behavior (*n* = 10)

Of the 10 studies assessing the effect of interventions on real world behavior, seven obtained and compared sales data prior to and following the implementation of a climate label in the university dining facility (30–34), two of which also included a non-randomized control dining facility for comparison (28, 29). The three other studies assessing the effect of interventions on actual behaviors utilized slightly different methods. Piester et al. surveyed students in line at the dining hall, randomly assigning them to view a menu online with or without the climate label and then collected receipts for participants' actual purchases (35). Castellanos et al. assessed the impact of a climate label intervention via post-intervention qualitative interviews with eight students (36). Zhao et al. assessed the impact of climate labels in three phases: (1) focus group discussions; (2) an experiment designed to emulate an auction to determine the cost consumers will pay for certain items; (3) an intervention in which three milk products were available in versions both with and without a carbon label (37) (Table 3).

Of these 10 studies, only Castellanos et al. reported no changes in consumer behavior based on qualitative interviews with eight students (36); the remaining nine studies found climate labels affected consumer behavior in at least some circumstances. Effect sizes varied substantially across studies. Among the nine studies that reported behavior effects, seven assessed the impact of climate labels on high-emissions food item sales, five assessed the impact on medium-emissions food item sales, and eight assessed the impact on low-emissions food item sales. Of the seven assessing high-emissions food item sales, one observed no change (30) and six observed reductions (28, 29, 31–34) in high-emissions food item sales. Of the five assessing medium-emissions food item sales, two observed no change (30, 32) and three observed increases (28, 29, 31) in medium-emissions food item sales. Of the eight studies assessing low-emissions food item sales, three observed no change (29, 32, 33) and five observed an increase in low-emissions food item sales (28, 30, 31, 35, 37) (Table 2).

#### 3.3.2 Online studies examining the effect of climate labels on hypothetical behaviors (*n* = 3)

Of the three online studies examining the effect of climate labels on hypothetical consumer behavior, two found that the labels affected consumer behavior (39, 41) and one found no effect (38). Suchier et al. utilized an experimental online grocery store to test the effect of two types of TLLs on hypothetical purchase behaviors: inter- and intra-TLLs (41). Inter-TLLs compare all foods in the store, while intra-TLLs compare foods within the same category. Notably intra- and inter-TLLs reduced purchasing of high-impact items and increased purchasing of low-impact items. Although the intra-TLL had a more substantial effect on high- and low-impact item purchasing, the kg CO_2_e/kg of participants' shopping baskets were identical (41). There is not sufficient evidence examining the potential difference between intra- and inter-TLLs in the climate label literature (20), Kimura et al. tested the effect of CO_2_e emissions quantity, CO_2_e emission information accessibility, and product type on willingness to pay and subjective ratings. Kimura et al. ultimately concluded that CO_2_e emissions quantity increased willingness to pay (39). Isham et al. tested willingness to pay and liking of plant-based products with either an ecological or health impact label and discovered that while the label had no effect on willingness to pay or liking, higher levels of life satisfaction were related to greater willingness to pay and greater liking of plant-based products (38) (Table 2).

#### 3.3.3 Studies assessing group-level heterogeneity in label effect (*n* = 5)

Five studies examined differences in label effectiveness by participant characteristics, three of which were intervention studies and two of which were online surveys. Across the five included studies, increased label effectiveness was observed for individuals who were female,^4^ (33, 35, 40) older (40), or white (40) in comparison to the other groups in the study, respectively (Table 3).

Of note, Piester et al. observed effects only for females (not males) (35), which is consistent with Isham et al. (38) and Brunner et al. (30) that also report that females generally follow a less emission intensive diet and are more responsive to labels. Slapo et al. observed effects only when a TLL was used (not a single red or single green label) (32). This was the only study that compared different label designs, although most studies that utilized a traffic light color scheme reported behavior changes.

In addition, Thamer et al. observed effects only when the climate label was paired with an email asking participants to reflect on the intervention or their dietary choices (34). Larner et al. observed effects only for certain items or when paired with a sales promotion (31), and Kimura et al. concluded that having consumers actively search for emissions information is associated with higher consumer valuation of the product (39). When taken together, these three studies suggest that additional interventions in the form of a sales promotion or a targeted reflection session can enhance the effects of climate labels.

## 4 Discussion

This paper provides a review of the use of climate labels to promote climate-friendly food choices within university settings. The literature included in this review suggests consumer behavior can, in certain circumstances, be swayed by climate labels in university environments. Among the studies analyzed, 86% (12 articles) observed a decrease in the consumption of foods researchers classified as high emissions, 60% (8 articles) observed a decrease in the consumption of foods classified as medium-emission foods, and 63% (9 articles) observed an increase in the consumption of foods classified as low-emission food. These findings are consistent with prior reviews, which have similarly found climate labels effective in some, but not all, situations (20, 42–44).

The existing literature is limited in its ability to identify best practices in climate labeling implementation and design. All the studies we identified had distinct label placement approaches and varying degrees of supplementary campaigns or messaging about the purpose of the climate label. No study formally tested the effectiveness of various placements, or specific messaging. There is also limited research comparing the efficacy of multiple labeling interventions or tests climate labeling in conjunction with additional interventions. For example, while some included studies also implemented an educational campaign to supplement the climate labels, more research is needed on the combined efficacy of the menu label supplemented by an educational campaign.

The studies included also used a wide variety of design elements (e.g., colors, images), but no clear trends emerged to inform which specific designs are most effective. The importance of label design has been established (20, 42–45). For example, when asked, adults indicate they prefer climate labels using scales (i.e., ordinal ratings) to nominal labels [labels with no clear intrinsic (ordering)] (46). Prior evidence also suggests simple single-metric labels (e.g., a scale displaying information on key environmental factors like carbon emissions or a simple overall eco-score) are effective in changing consumer behaviors (47). Yet only two studies directly compared the effectiveness of different label designs with mixed findings. Further research comparing specific label designs, intra- vs. inter- label categories, positive vs. negative framing, label placement, and accompanying marketing campaigns is needed to aid institutions in understanding best practices for designing and implementing climate labels.

Only five of the included studies included stratified or effect modification analyses examining the difference in climate label effect by key factors (e.g., income, gender, student status, psychological factors); labels were more effective for individuals who were female, (33, 35, 40) older (40), white (40), followed a high-carbon footprint diet (29), and had higher levels of wellbeing (38). While these studies provide insight into how implementers might target certain groups to realize consumer behavior changes, further research may provide additional evidence to support existing studies or examine groups not already considered (e.g., ethnicity). For example, studies should also investigate the role ethnicity plays in impacting food preferences and purchases.

In addition, among the studies that considered impact of labeling foods as “low,” “medium,” or “high” emissions, studies that included food labeled “high emission” noted the greatest change in consumer demand. Thus, it is possible that labeling a product as “high emissions” has the largest effect on consumer behavior, suggesting consumers respond stronger to negative framing than positive framing. However, additional research is required to validate this theory.

The studies reviewed generally evaluated the impact of climate labels over a few weeks or months. Prior research in other settings suggests repeated exposure increases climate label effectiveness, though their impact may diminish over time (48). Investigating this in university settings could deepen our understanding of label effectiveness. Additionally, researchers may consider examining how climate labels influence food purchases beyond the university setting, such as when students eat out, buy groceries off campus, travel home, or post- graduation, as well as potential spillover effects on friends and family. Studying the spill-over impact of climate labels will help researchers understand the broader potential for such labels to change behavior on a larger scale.

The included studies focused on outcomes related to liking, willingness to pay, purchasing, or sale of food items; however, if climate labels change consumer behavior, they may also impact other important factors, such as the healthiness of food products consumed or food waste. Prior research has suggested labeling products as eco-friendly may increase individuals' perceptions of healthfulness (49); yet, another study using an online survey to examine the hypothetical effect of climate labels in fast food restaurants showed consumers chose meals that were not only more sustainable, but also healthier (50). Such research specific to university settings would provide valuable insight into the implementation of climate labels in these settings. For example, if climate labels increase the healthiness of food consumed, it may be possible to garner additional support for and interest in implementing climate labels. On the other hand, if they reduce the healthiness of food consumed, further research could explore ways to mitigate such an effect.

None of the included studies examined the upstream effects of climate labels on institutional purchasing or production, though the goal of climate labeling is likely closely tied to these components. If minor shifts in sales occur, it is possible that institutional purchasing would not change in a manner meaningful enough to reduce the GHG emissions of purchased foods. Even if institutional purchasing did change in a manner that reduced GHG emissions, it is not clear if/how actual sourcing or production practices would change.

### 4.1 Strengths and limitations

This narrative review is strengthened by the systematic protocols employed, and the breadth of databases searched. However, there are several key limitations to this literature review. Notably, the included articles were not formally reviewed for quality as would be done in a systematic literature review. While detailed information was included about each article to allow readers to get a sense of the quality, the included studies were potentially subject to issues with sampling bias, information bias, or insufficient power, which we did not formally assess. Thus, while the protocol used was intended to describe a complete picture of the existing literature regarding climate labels in university settings, it is not intended to and should not be read as a definitive determination as to the effect of climate labels on consumer behavior in university settings. A systematic literature review that includes quality control may be warranted, particularly as the literature in this field of research continues to develop. Additionally, gray literature was not included in this search. It is possible such literature may have additional and/or different information than the included academic articles.

In addition, variations in study design, location, data collection years, time frames, label designs, or label implementation may contribute to the inconsistent effects observed. Additionally, some of the current studies may have been underpowered given their small sample sizes; notably a few studies included less than 100 participants. Future studies may consider including a larger sample of individuals and/or dining halls and formally testing how the factors mentioned above (e.g., label design, time frame) alter label effectiveness.

Furthermore, universities provide controlled and semi-structured environments where the student population remains relatively stable over academic terms. This setting allows researchers to track changes in behavior, enabling longitudinal studies of how climate labels influence purchasing decisions and consumption patterns. While university students offer a convenient and consistent sample, their behaviors may not fully represent the broader population. Students tend to be younger, more educated, and often more environmentally conscious than the public, potentially skewing the results and limiting the external validity of findings. Thus, while this study may provide some guidance on the use of climate-labels outside of this context, additional research will be required to validate the generalizability of our findings.

## 5 Conclusion

The current literature suggests climate labels can be an effective way to shift consumer behaviors in university settings away from food items associated with high GHGE and/or toward food items associated with low GHGE in certain circumstances. The key patterns that emerged during this review include (1) a small, yet detectable behavior shift in consumer behavior with the introduction of labeling, (2) negative framing as a more powerful tool in comparison to positive framing, and (3) women appear to have a more significant response to climate labels. There are several key gaps in the existing literature, including but not limited to: (1) how to improve the effectiveness of climate labels (e.g., via label design and/or concurrent complementary interventions and/or messaging campaigns), (2) how the effect of climate labels may vary by subgroup (e.g., income, race, ethnicity, psychological factors), (3) the longer-term and/or spillover effects of climate labels on climate-related outcomes (e.g., the healthiness of consumer diets, group/social dynamics, and broader institutional sourcing practices). While these gaps need to be addressed in subsequent research, this review has demonstrated that climate labels can be a useful tool for helping consumers shift their dietary behavior.

## Footnotes

1 While there are other metrics for environmental sustainability (e.g., water pollution, soil health, and land use), GHGE is one of the most widely used based on its direct link to climate change and alignment with environmental policies (2).

2 CO_2_e is a standard unit for measuring the global warming potential of different greenhouse gases, that expresses the impact of various greenhouse gases in terms of the amount of carbon dioxide (CO_2_) that would produce the same warming effect.

3 ENERGY STAR Impacts | ENERGY STAR [Internet]. [cited 2025 Mar 27]. Available online at: https://www.energystar.gov/about/impacts.

4 A note about terminology: throughout this paper, the authors utilize the same demographic language as the source papers.
