# Supplementary material for: Implementing climate menu labels in university settings: a narrative review
Source: Front Nutr. 2025 Nov 21;12:1619842. doi: 10.3389/fnut.2025.1619842 (PMC12679830; doi:10.3389/fnut.2025.1619842)
Supplement: Supplementary file 1 [file Table_1.docx]

**Supplementary Material**

**Table 1S: Overview of data extracted from included articles**

**Appendix 1. Overview of data extracted from included articles**

| CATEGORY | SUBCATEGORY | FIELD |
| --- | --- | --- |
| STUDY  DETAILS | STUDY IDENTIFICATION | Citation |
|  |  | Abstract |
|  | LOCATION | Country |
|  |  | School |
|  |  | Setting |
|  | STUDY TYPE | What type of study was done to test the author’s hypothesis? |
|  | DATA COLLECTION DATES | What was the study duration? |
|  | SAMPLE | What was the sample unit? |
|  |  | What was the sample size? |
|  |  | Was there any stratification in analysis? |
|  |  | What was the demographic breakdown of the sample? |
|  | AIM | What was the goal of labeling? |
| LABELS | NAME | What were the labels referred to as? Was this discussed / tested on its own? |
|  | DEFINITION & DESIGN | What do the labels represent? |
|  |  | How were they calculated? |
|  |  | How are they broken down? |
|  |  | What color scheme did the labels use? |
|  |  | What did the labels look like? |
|  |  | What was the thought process / justification of the labels? |
|  | PLACEMENT | What was labeled? |
|  |  | Where did the physical labels appear in reference to the food? |
|  | IMPLEMENTATION | Was there any messaging about the intervention (e.g., to convey label function)? |
| OUTCOMES | METRICS | What tools were used to measure label impact during the study? |
|  | RESULTS & ANALYSIS | What were the overall results/conclusion of the study? |
|  |  | Did some sub-groups adhere more than others? |
|  | LIMITATIONS | What challenges / limitations were experienced? How were they addressed? |
